# Unexplained recurrent shock in peripheral T‐cell lymphoma: A case report

**DOI:** 10.1002/ccr3.4612

**Published:** 2021-08-10

**Authors:** Hiroshi Imamura, Yuichiro Kashima, Masao Hattori, Kotaro Mori, Kanako Takeshige, Hideyuki Nakazawa

**Affiliations:** ^1^ Department of Emergency and Critical Care Medicine Shinshu University School of Medicine Matsumoto Japan; ^2^ Department of Hematology Shinshu University School of Medicine Matsumoto Japan

**Keywords:** case report, peripheral T‐cell lymphoma, shock, tracheobronchomalacia

## Abstract

Malignant lymphoma sometimes manifests with septic‐like shock symptoms. We report a case of peripheral T‐cell lymphoma presenting with unexplained recurrent shock in absence of apparent lymphadenopathy. The patient also experienced varied symptoms, including severe chest and back pain, respiratory distress due to tracheobronchomalacia, skin rash, and fever.

## Graphical abstract

Malignant lymphoma sometimes manifests with septic‐like shock symptoms. We report a case of peripheral T‐cell lymphoma presenting with unexplained recurrent shock in the absence of apparent lymphadenopathy.

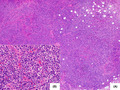

## 1 INTRODUCTION

Malignant lymphoma may be responsible for various life‐threatening conditions, including pulmonary dysfunction or acute circulatory failure.^1^ Herein, we present a case of peripheral T‐cell lymphoma, not otherwise specified (PTCL‐NOS), in a patient who presented with unexplained recurrent shock and severe chest and back pain, in the absence of apparent lymphadenopathy.

## 2 CASE PRESENTATION

A 70‐year‐old man with shock and severe chest and back pain experienced exanthema with itching on his legs 4 weeks before admission, which spontaneously disappeared after 1 week. Before admission, the patient also experienced sore throat for 3 weeks. On the day of admission, he experienced abrupt chest and back pain and loss of consciousness. His wife noted that he appeared pale and had cold sweats. The patient regained consciousness after his wife tapped him on the back. When emergency medical technicians arrived, the patient was alert but in shock, with a pulse rate of 77 bpm, blood pressure of 63/52 mmHg, and respiratory rate of 24 breaths per min. Ringer's solution was administered, and his systolic blood pressure increased to 100 mmHg.

The patient's medical history included subarachnoid hemorrhage 3 years prior to admission, which was treated with coil embolization and placement of a ventriculoperitoneal shunt, early‐stage gastric cancer, resected endoscopically using submucosal dissection 2 years before admission, and tracheostomy that was performed in conjunction with a neurovascular surgery and closed thereafter. His medications included atenolol, atorvastatin, lansoprazole, and rivaroxaban. Regarding the patient's social history, he lived at home and received care from relatives. He was a former smoker and social drinker with no history of illicit drug use. He had no family history of malignancy or autoimmune/inflammatory disease.

Once he arrived at the hospital, he was alert with no complaints of chest or back pain. Moreover, his vital signs were stable with a pulse rate of 71 bpm, blood pressure of 101/47 mmHg, respiratory rate of 18 breaths per min, and temperature of 36.4℃. His oxygen saturation was 100% with administration of 5 L/min O_2_.

Physical examination revealed no jugular vein distension. The lungs were clear with normal cardiac findings. In addition, no superficial lymphadenopathy was noted. Electrocardiography revealed non‐specific ST‐T segment abnormality. Chest radiography showed no abnormalities. His initial laboratory findings were as follows: decreased hemoglobin level (12.7 g/dl); normal white blood cell count (5.10 × 10^9^ cells/L); normal platelet count (193 × 10^9^ cells/L); normal serum creatinine (1.01 mg/dl) level; normal total bilirubin (0.43 mg/dl) level; normal electrolytes and acid‐base balance; increased aspartate aminotransferase (61 U/L), alanine aminotransferase (90 U/L), lactate dehydrogenase (518 U/L), alkaline phosphatase (748 U/L), and λ‐glutamate transpeptidase (203 I/L) levels; slightly increased C‐reactive protein (1.03 mg/dl) level; and normal troponin (0.010 ng/ml) and venous lactate (2 mmol/L) levels. His glucose level was 144 mg/dl. In addition, his serum free triiodothyronine, free thyroxine, and thyrotropine levels were 2.08 pg/ml, 1.14 ng/dl, and 1.19 μIU/L, respectively. Blood tests for human immunodeficiency virus (HIV) type 1 antigen, antibodies to HIV types 1 and 2, and antibodies to human T‐lymphotropic virus type 1 were negative.

**FIGURE 1:**
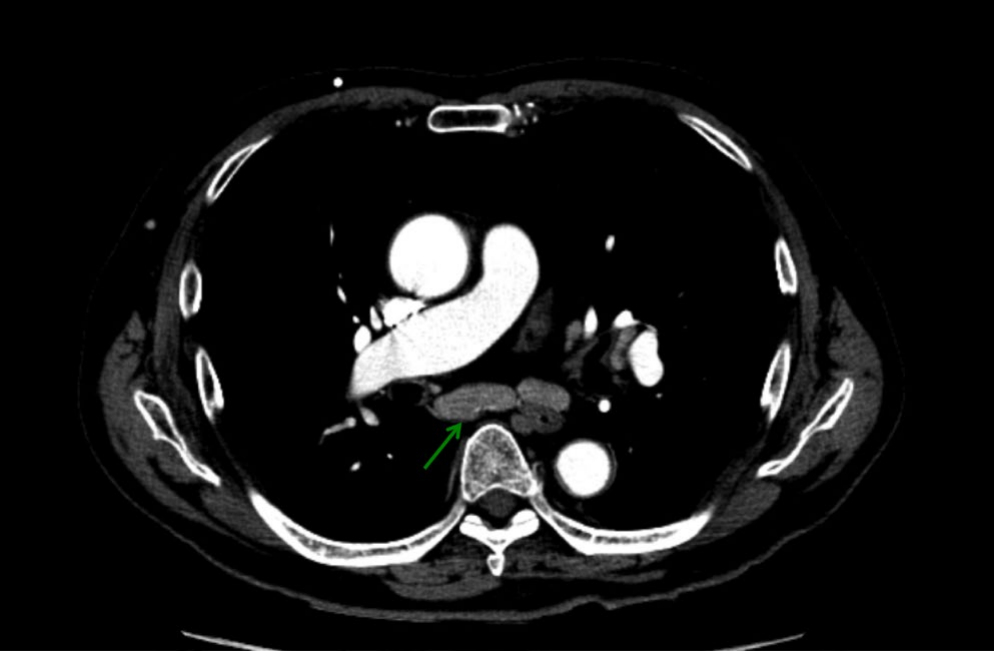
Post‐contrast axial chest computed tomography. Cardiovascular abnormalities are not observed. The subcarinal lymph nodes (green arrow) are slightly enlarged

Transthoracic echocardiogram revealed no left ventricular segmental asynergy. Chest and abdominal computed tomography (CT) did not detect any signs of aortic dissection, pulmonary embolism, or biliary disease; however, borderline mediastinal lymphadenopathy was noted (Figure 1). Coronary angiography indicated no substantial stenosis.

There was no evidence of cardiogenic, hypovolemic, and obstructive shock. In addition, no evidence of endocrine and metabolic diseases was found. The patient was admitted to the hospital because of shock without a clear etiology and was observed closely. After admission, he was stable with no symptoms, except for complaints of sore throat. Laryngoscopy was performed to further evaluate this symptom, which showed normal findings. On day 4, his temperature increased to 39.7℃, and his blood pressure decreased to 60 mmHg. Laboratory test results demonstrated a white blood cell count of 6.350 × 10^9^ cells/L and a C‐reactive protein level of 6.01 mg/dl. Following crystalloid, noradrenalin, and tazobactam/piperacillin administration for suspected sepsis, his blood pressure and temperature normalized. His blood culture was negative for pathogens. On day 14, he developed fever again (39℃). Soft, non‐tender lymph nodes were palpable bilaterally at the anterior neck and supraclavicular region. Tazobactam/piperacillin treatment was then withheld, and a more in‐depth laboratory evaluation was performed. The patient's antinuclear antibody was positive (1:2560, homogeneous and nucleolar pattern). Anti‐double‐stranded DNA, anti‐RNP, and anti‐CL IgG antibodies were slightly elevated (16.5, 14.9, and 14 U/ml, respectively). Serologic studies of neutrophil cytoplasmic, anti‐Sm, SSA, SSB, Scl, and Jo‐1 antibodies were normal. C3 and C4 levels were slightly low (53 and 8.2 mg/dl, respectively). Soluble IL‐2 receptor level was elevated (3299 U/ml). The patient denied arthralgias, butterfly rash, mouth ulcers, alopecia, or photosensitivity.

**FIGURE 2:**
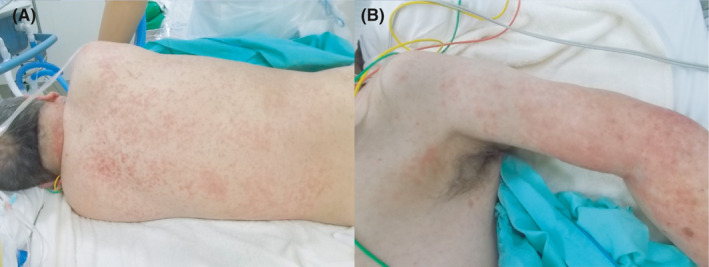
Photographs of skin rash on the back (A) and the left arm (B) on day 21

**FIGURE 3:**
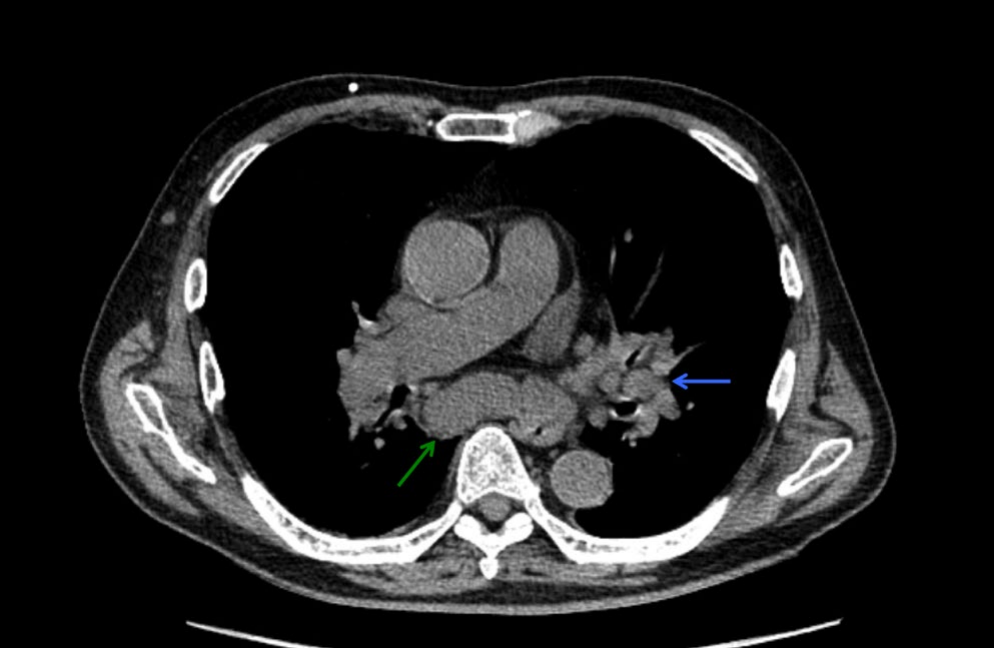
Chest computed tomography on day 21. Marked enlargement of the subcarinal (green arrow) and hilar (blue arrow) lymph nodes observed

On day 21, he developed hypotension (blood pressure, 83/60 mmHg), respiratory distress (respiratory rate, 37 breaths per min) with his oxygen saturation maintained at 97% on 2 L/min O_2_, generalized skin rash (Figure 2), and high fever (41℃). Marked expiratory wheeze was detected and did not improve with bronchodilator therapy. Chest and abdominal CT showed no pulmonary infiltrates but revealed enlarged mediastinal and abdominal lymph nodes (Figure 3). The patient was intubated, and noradrenaline was administered. Bronchoscopy revealed collapsed trachea and mainstem bronchi during expiration and edema of the tracheobronchial wall.

**FIGURE 4:**
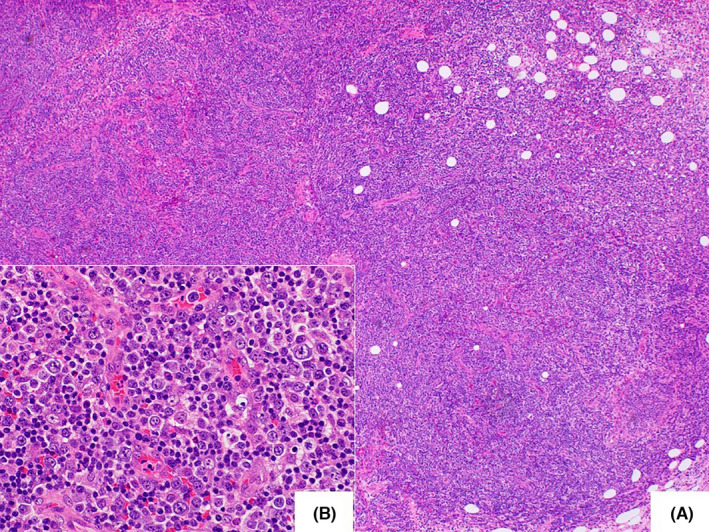
Cervical lymph node biopsy. Biopsy reveals complete effacement of the normal architecture and diffuse proliferation of large lymphoid cells with vascular proliferation (hematoxylin and eosin staining, original magnification 40× [A], 400× [B])

**FIGURE 5:**
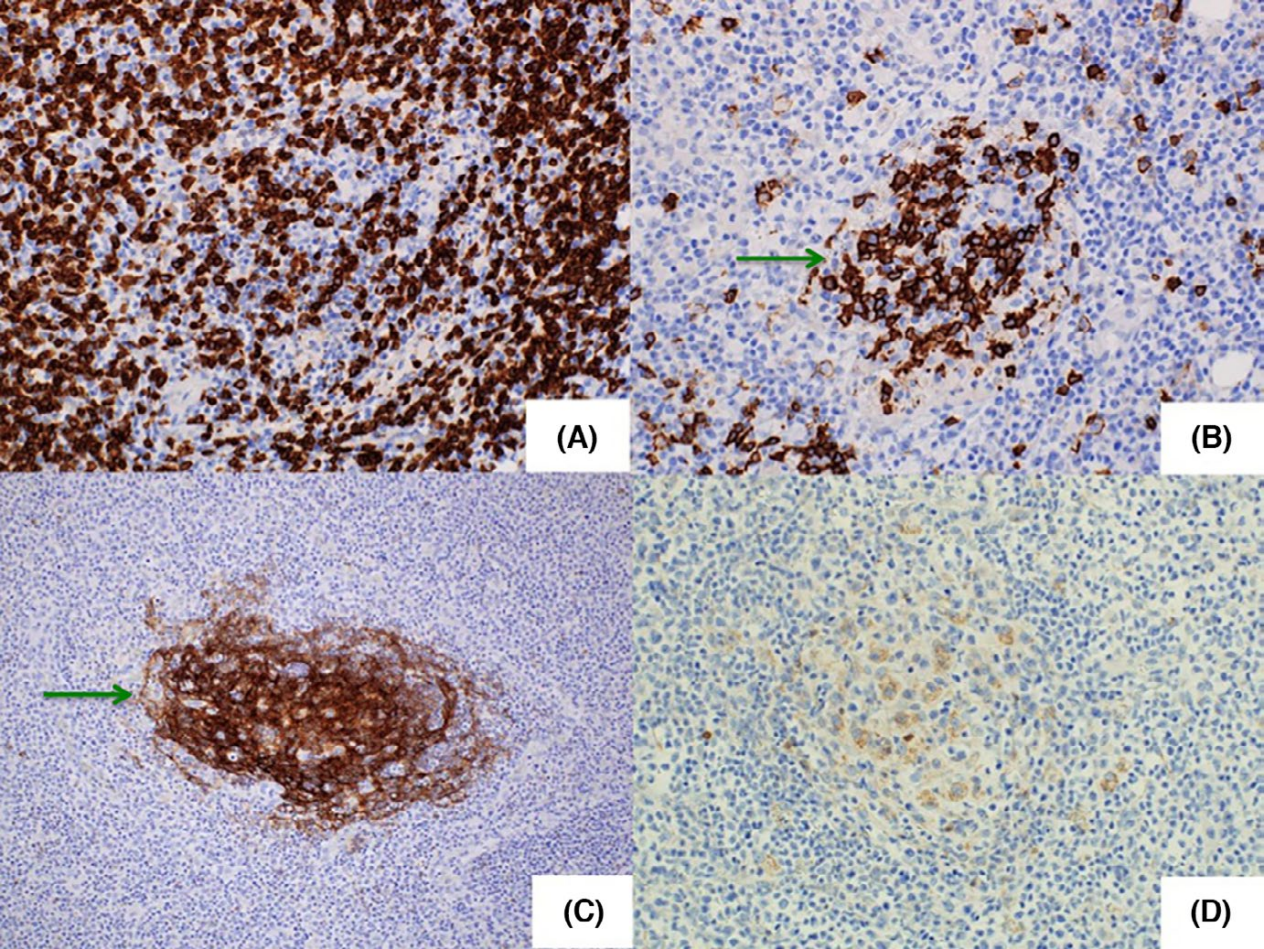
Immunohistological examination. (A) The tumor cells are positive for CD3 (original magnification 200×). (B) CD20‐positive cells are scarce but found within residual follicles (green arrow, original magnification 200×). (C) CD21‐positive cells are limited within residual follicles (green arrow, original magnification 100×). (D) Tumor cells are negative for CD10 (original magnification 200×)

**FIGURE 6:**
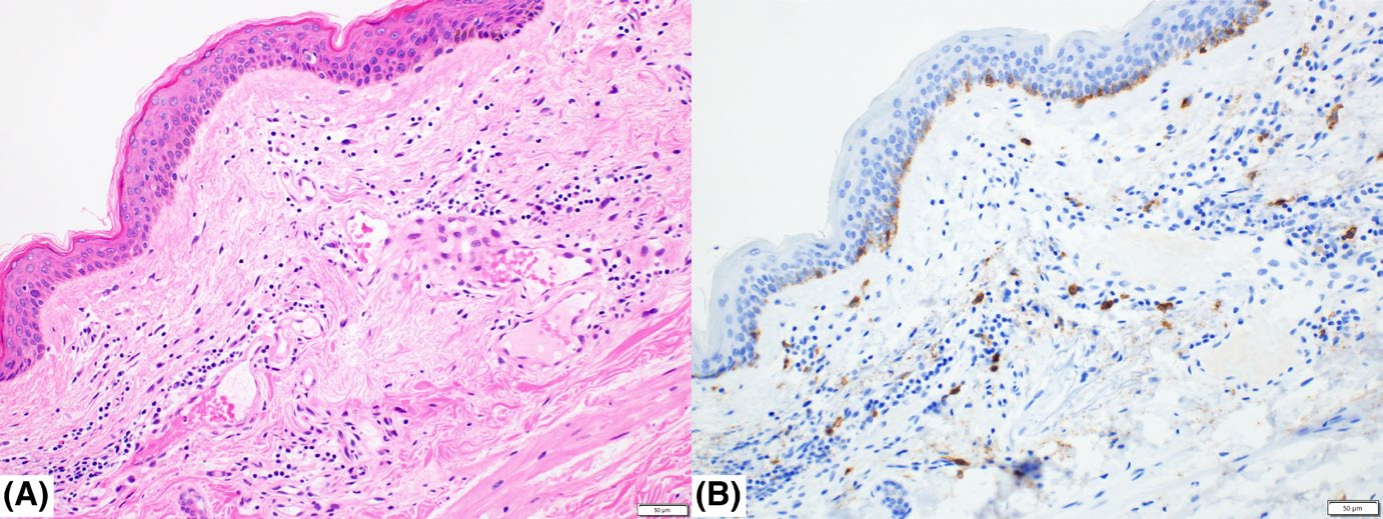
Skin biopsy. There is a small amount of perivascular infiltration of small lymphocytes. The lymphocyte morphology differs from that of large lymphoid cells in the lymph node. There is no infiltration of mast cells (A). CD117 is negative (B)

Cervical lymph node biopsy performed on day 23 revealed complete effacement of the normal architecture, diffuse proliferation of tumor cells, and some vascular proliferation (Figure 4). Unlike those in angioimmunoblastic T‐cell lymphoma (AITL), the tumor cells were rather large. These tumor cells were positive for CD3 and CD5, and CD4 was dominant. They were negative for CD10 and Bcl‐6 and only sporadically positive for CXCL13 and PD‐1. There was no dendritic cell proliferation, and the CD21‐positive cells were limited within residual follicles, which were CD20 positive. Tumor cells were negative for TIA‐1, positive for Granzyme B, and sporadically positive for CD56. A small number of EBV‐ISH‐positive cells were also observed. These findings confirmed a diagnosis of peripheral T‐cell lymphoma, not otherwise specified (PTCL‐NOS, Figure 5).^2^, ^3^, ^4^ Skin specimens obtained at the time of lymph node biopsy indicated a small amount of perivascular infiltration of small CD3‐positive lymphocytes (Figure 6). The lymphocyte morphology differed from that of large lymphoid cells in the lymph node. There was no infiltration of mast cells and CD117 was negative. Thus, a diagnosis of systemic mastocytosis was excluded. Bone marrow biopsy showed bone marrow hypoplasia. There was a small infiltration of CD3‐positive atypical cells, which suggested that the disease stage was IV.

Treatment with prednisolone, 60 mg/day, was initiated on day 23, and his condition improved the following day. His temperature and heart rate decreased, and blood pressure increased. Noradrenaline was discontinued on day 24. He was extubated on day 27, and non‐invasive positive pressure ventilation (NIPPV) was performed. His expiratory wheeze gradually improved thereafter. The patient was weaned from NIPPV on day 35. At this time, he could eat and walk unassisted. Chemotherapy was not performed due to his overall poor performance status. The prednisolone dose was tapered starting on day 38, without any subsequent exacerbation. He was transferred to another hospital for rehabilitation on day 45. Thirty months later, complete remission was achieved, and the patient is currently on prednisolone (10 mg per day).

## 3 DISCUSSION

PTCL‐NOS is the most common PTCL subtype, accounting for ≥25% of all PTCL cases.^4^, ^5^ It predominantly affects adult men (median age of onset: 60 years). Clinically, this present patient had some features of AITL, including fever, skin rash, positive autoimmune test, and relatively small lymph nodes,^6^, ^7^ while he also had a history of repetitive episodes of hypotension and systemic skin rash often encountered in systemic mastocytosis. However, pathologically, PTCL‐NOS was diagnosed on an exclusion basis as a disease whose histological features were not consistent with any of the other pathological entities defined by the World Health Organization classification.^2^, ^8^

Malignant lymphoma rarely causes acute circulatory failure that can mimic septic shock.^1^, ^9^, ^10^ The mechanisms for shock may involve an overwhelming production of proinflammatory cytokines by malignant cells and/or activated macrophages. This patient's case was atypical as he developed severe circulatory shock with no apparent lymphadenopathy. Moreover, he experienced significant chest and back pain of unknown origin mimicking an acute coronary or aortic syndrome. Diagnosis of malignant lymphoma was suspected due to the high fever of non‐infectious origins, laboratory findings consistent with autoimmune phenomena, and gradual enlargement of lymph nodes. It might be possible that the cervical lymph nodes and the inflammation around them could have evoked sympathetic and parasympathetic nerve reaction, which might have caused further hypotension on day 21.

The patient exhibited skin rash several times before and after admission. Systemic mastocytosis was excluded based on the skin biopsy. Skin specimens indicated a small amount of perivascular infiltration of small CD3‐positive lymphocytes. The lymphocyte morphology differed from that of large lymphoid cells in the lymph node. Therefore, reactive perivascular dermatitis was suspected.

Our patient experienced severe respiratory distress due to tracheobronchomalacia and bronchial edema.^11^, ^12^ His tracheobronchomalacia before admission might have occurred due to his smoking and tracheostomy history. However, an expiratory wheeze was not heard until day 20. Capillary leakage and/or infiltration of malignant cells might have caused edema and bronchial stenosis, leading to this finding.

## 4 CONCLUSION

We describe a patient with PTCL‐NOS who experienced recurrent circulatory shock with no apparent initial lymphadenopathy. He also experienced varied symptoms, such as severe chest and back pain, respiratory distress due to tracheobronchomalacia, skin rash, and fever. Therefore, patients with related presenting findings should undergo an in‐depth evaluation to rule out underlying malignant causes. A detailed review of case reports of patients with shock who were later diagnosed with cancer would be helpful to support this conclusion.

## CONFLICTS OF INTEREST

The authors of this work have nothing to disclose.

## AUTHOR CONTRIBUTIONS

HI: Wrote the first draft of the article. YK, MH, and KT: Managed the patient. KM: Analyzed the pathologic pictures and revised the manuscript. All authors read and approved the final manuscript.

## ETHICAL APPROVAL

The patient has provided written informed consent for the publication of this case report.
